# Electrospun PLGA Membranes with Incorporated Moxifloxacin-Loaded Silica-Based Mesoporous Nanocarriers for Periodontal Regeneration

**DOI:** 10.3390/nano12050850

**Published:** 2022-03-02

**Authors:** Georgia K. Pouroutzidou, Maria Lazaridou, Chrysanthi Papoulia, Ioannis Tsamesidis, Konstantinos Chrissafis, George Vourlias, Konstantinos M. Paraskevopoulos, Dimitrios Bikiaris, Eleana Kontonasaki

**Affiliations:** 1 Advanced Materials and Devices Laboratory, Faculty of Sciences, School of Physics, Aristotle University of Thessaloniki, 54124 Thessaloniki, Greece; gpourout@physics.auth.gr (G.K.P.); cpapouli@physics.auth.gr (C.P.); hrisafis@physics.auth.gr (K.C.); gvourlia@auth.gr (G.V.); kpar@auth.gr (K.M.P.); 2 Department of Prosthodontics, Faculty of Health Sciences, School of Dentistry, Aristotle University of Thessaloniki, 54124 Thessaloniki, Greece; johntsame@gmail.com; 3 Faculty of Sciences, School of Chemistry, Aristotle University of Thessaloniki, 54124 Thessaloniki, Greece; marlazach@chem.auth.gr

**Keywords:** electrospun membranes, mesoporous nanoparticles, drug delivery, MCM-41, moxifloxacin, PLGA, electrospinning process, sol-gel technique, periodontal restoration, hemocompatibility

## Abstract

Engineered electrospun membranes have emerged as promising materials in guided tissue regeneration, as they provide an appropriate framework for the formation of new functional periodontal tissues. The development of multifunctional local drug delivery systems with sustained release of drugs for prolonged infection control can be used in periodontal surgical interventions to simultaneously prohibit epithelium downgrowth and ensure proper healing and regeneration of damaged periodontal tissues. The aim of the present study was the fabrication of novel composite membranes from PLGA/moxifloxacin-loaded mesoporous nanocarriers through electrospinning and the evaluation of their drug release profiles. The addition of moxifloxacin-loaded mesoporous nanocarriers in PLGA yielded a sustained and prolonged drug release, while maintaining satisfactory mechanical strength. The freshly fabricated membranes were found to be biocompatible at masses less than 1 mg after exposure to healthy erythrocytes. Increase in the amount of polymer led to more uniform fibers with large diameters and pores. The study of the parameters of the electrospinning process indicated that increase in the applied voltage value and rotation speed of the collector led to more uniform fibers with higher diameter and larger pores, suitable for tissue regeneration applications, such as periodontal tissue regeneration.

## 1. Introduction

Periodontitis is a multifactorial, multifaceted chronic inflammatory condition that affects the periodontium (the soft and hard tissues that surround the tooth) causing irreversible loss of tooth retention. The failure to diagnose and treat the disease can lead to severe damage to the alveolar bone and tooth support causing functional alterations that could lead to premature tooth loss. The consequences of untreated periodontal disease have a wide range of effects on a person’s quality of life. Thus, the goal of periodontal restoration is to maintain and/or regenerate the architecture of the dental support system in order to maintain its function [1,2]. A wide variety of treatments have been proposed to treat different stages of the disease, starting with simple receding gingival tissue and ending with more aggressive periodontal infection associated with bone breakdown [1,2].

Within this framework, guided tissue regeneration (GTR) was introduced as an effective strategy for the treatment of periodontal infections [3]. This approach involves the complete reconstruction of the injured tissue using synthetic barrier membranes to prevent epithelial downgrowth, providing an appropriate healing space for bone and periodontal tissue [3,4]. Bioabsorbable membranes are considered the most suitable candidates considering the lack of need for a second surgery, which guarantees a lower chance of infection [3,4]. Attempts to provide regenerative therapies in periodontitis, such as guided tissue/bone regeneration (GTR/GBR), for periodontal regeneration have also been performed [5,6]. In this respect, membranes that act as scaffolds, simulating the extracellular matrix (ECM), could be more effective in recruiting progenitor cells from the surrounding tissues that could differentiate into osteoblasts, fibroblasts, and cementoblasts, which are able to regenerate the complete periodontal tissue apparatus [5,6].

Biodegradable polymers with high processing flexibility are the most sought-after candidates for tissue engineering applications. Synthetic biodegradable polymers with a well-defined structure and without immune reactions widely used in tissue engineering include polyesters, polyhydrates, polyurethanes, etc. [7]. The most widely used synthetic biodegradable polymers in tissue regeneration are aliphatic polyesters. Aliphatic polyesters can form stable porous materials that do not dissolve or melt in vitro and are used as 3D scaffolds. Such materials are regularly degraded by the hydrolysis of esters. Degradation rates and degradation products can be adjusted according to composition, structure, and molecular weight. Poly(lactic acid) (PLA), polyglycolic acid (PGA), and their copolymer poly(lactic-glycolic acid) (PLGA) are some of the well-known polyesters [7,8,9]. PLGA is a synthetic copolymer, approved by the US Food and Drug Administration for medical applications. PLGA degradation rates can be adjusted to range from weeks to months, based on the ratio of PLA to PGA within the copolymer structure [10,11,12]. The range of commonly available solvents in which PLGA can be dissolved and the ease with which it can be formed in desired sizes and shapes are two of the many advantages of PLGA over pure PGA and PLA. Biomolecules, such as active substances and growth factors, can also be easily encapsulated by PLGA [10,11]. PGA is more hydrophilic than PLA due to the lack of additional asymmetric methyl groups. Therefore, the addition of PGA to a PLGA copolymer is a method of increasing the water solubility of PLGA. Another consequence of the hydrophilicity of PGA is its relatively fast degradation rate. PGA is degraded in about two to four weeks in vivo, losing up to 60% of its mass in the first two weeks. Therefore, the appropriate PLA/PGA ratio could give the scaffolds the desired rate of degradation with enhanced mechanical properties [13]. PLGA, has been employed for periodontal regeneration because of its favorable mechanical properties, controllable degradation rates, and improved biocompatibility. Despite its appealing characteristics, PLGA presents constraints related to its hydrophobicity and restricted bioactivity. Improving these functions of the polymer could make it appropriate in applications needing barrier membranes, bone grafts, and drug transport carriers, which implies that PLGA may be an excellent candidate in periodontal regenerative medicine [14].

The application of the systemic administration of growth factors and/or drugs for the enhancement of bone regeneration is accompanied by significant side effects and limited bioavailability [15,16]. Although various local release systems of drugs or growth factors are being investigated, controlled, and sustained release for the extended periods of time required for new tissue formation has not been achieved yet. Therefore, the most important challenge of a composite scaffold for bone regeneration is to additionally provide the possibility of local, controlled dissolution/release of growth factors in a normal environment, to effectively induce its osteogenetic and angiogenic capacity [15,16]. Composite bioceramic/polymer scaffolds could incorporate growth factor carriers that contribute to osteogenesis. Silica-based mesoporous nanocarriers (MSNs) synthesized via the sol-gel technique, which produces high purity homogeneous products by controlling particle size, distribution, and morphology, could improve not only the mechanical properties of composite scaffolds for periodontal tissue regeneration, but also the capacity for apatite formation and the release of various drugs and molecules [17,18,19,20,21,22,23,24,25,26,27]. The use of MSNs doped with calcium Ca and/or magnesium Mg ions, as well as strontium Sr, can provide a dynamic carrier system, with appropriate surface structure, chemical stability, osteoinductive action, and appropriate release profile. However, due to the problems of inflammation during the early stages of implantation, there is a need for alternative approaches to the antibiotics used today [28].

Moxifloxacin (MOX), ciprofloxacin, levofloxacin, and ofloxacin are alternative antibiotics. They belong to the fourth generation of fluoroquinolone antibiotics and provide antimicrobial activity against a wide range of aerobic and anaerobic bacteria, including *S. aureus*, which is the major pathogen associated with osteomyelitis. MOX is superior to ciprofloxacin, levofloxacin, and ofloxacin against *Staphylococcus aureus,* and could therefore be applied locally for bacterial eradication [29]. Moxifloxacin exerts superb antibacterial activity against an extensive variety of putative periodontal pathogens, such as *Porphyromonas gingivalis*, *Tannerella forsythia*, *Peptostreptococcus* spp., etc. Its bactericidal effectiveness in opposition to biofilm-embedded *Porphyromonas gingivalis*, *A. actinomycetemcomitans*, and *Streptococcus constellatus* was observed to be advanced compared to other drugs. Moxifloxacin penetrates effectively into the tissues and its systemic administration in combination to scaling and root planing proved more efficient compared with doxycycline or scaling and root planing alone [30,31,32,33,34,35].

In tissue engineering, scaffolds or membranes come in direct contact with living tissues, with blood being the first. At the blood–biomaterial interface, several physicochemical characteristics can cause surface-induced thrombosis or hemolysis, through erythrocyte membrane rupture and local release of hemoglobin [36]. Hemolysis can affect bone regeneration, primarily by affecting the healing process. Bone healing occurs through blood clot stabilization and hematoma dissolution, which results in the release of chemotactic agents, such as cytokines and growth factors that induce the recruitment of osteoprogenitor cells [37], which proliferate and differentiate to produce mature bone forming cells [38]. For periodontal regeneration, new alveolar bone should be formed along with cementum and periodontal ligament [38]. Thus, bone healing and regeneration is ensured with engineered constructs with blood compatible surfaces, rendering hemocompatibility an important factor in materials design for tissue engineering applications [39,40,41,42].

The creation of a nanostructured scaffold matrix that mimics bone tissue architecture is a major challenge limiting its clinical application to date [43]. Within this framework, electrospinning is a potential technique that has been actively explored recently, due to its simple process which offers nanosized polymer fibers with high specific surface area, and the possibility of various modifications to create biomimetic nanofibrous membranes which can provide an excellent microenvironment for cell adhesion, migration, proliferation, and differentiation [44,45,46]. In periodontal tissue engineering, recent studies have highlighted the effectiveness of electrospun matrixes to support cell loading, proliferation and differentiation and in vivo periodontal tissue regeneration, in combination with growth factors or cell seeding. Ding et al., created core/shell composite PLLA/PLGA fibrous scaffolds through electrospinning for the dual release of basic fibroblast growth factor (bFGF) and bone morphogenetic protein-2 (BMP-2) [47]. After eight weeks of implantation in intrabony defects into the alveolar bone crest of Wistar rats, they observed the regeneration of the whole periodontal apparatus (bone, cementum, and periodontal ligament (PDL)). Similar results were obtained by Cai et al., that used a bone-marrow-stem-cell-loaded PLGA-PCL electrospun scaffold in a periodontal defect model in the Fisher rat and reported newly formed trabecular bone, cementum and obliquely oriented PDL formation after six weeks of implantation [48].

Electrospinning is a controlled and reliable method for designing and generating scaffolds intended for tissue engineering applications [6,49,50,51,52]. One-dimensional nanostructures or nanofibers have been the subject of intensive research due to their unique properties and interesting applications in various fields, such as bone tissue regeneration [53,54]. The main principle behind the formation of very fine fibers by electrospinning is based on the elongation of a viscoelastic jet coming from a polymer solution or melt [54]. A typical power supply device consists of three main parts: the high voltage supply (kV), the metal needle, and the ground collector [51]. In a typical laboratory electrospinning experiment, a polymer solution feeds the generated jet through a syringe that ejects the solution at a controlled rate. The tip of the needle has the use of an electrode to which a high voltage electric field is applied. The high voltage which is applied to the solution must be over a critical value, typically higher than 5 kV, so that the repulsive force within the homogeneously charged solution is higher than its surface tension such that a jet can be formed which will be ejected from the tip of the needle. Generally, a grounded target can act as an oppositely charged electrode which is used to collect the resulting fibers, either in rotating or static collectors [51]. In a laboratory setting, the distance of the needle and the oppositely charged electrode is usually 8–25 cm. The applied voltage causes a deformation in the shape of the falling cone of the polymer solution, in the direction of the opposite electrode. As the solvent reaches the opposite electrode and evaporates, solid fibers with diameters in a range of μm to nA are formed at high velocities at the opposite electrode (collector) [55]. The technique of electrospinning is related to the interaction of many physical instability processes. The jet which is formed during the electrospinning process consists of four areas: the base, the main jet, the grid, and the collection. In the area of the base, the jet comes out of the needle to form a cone known as the Taylor cone. The shape of the base depends on the surface tension of the solution and the strength of the electric field. If the electric field is strong enough, the jets can be ejected from surfaces that are substantially flat [56].

In the process of electrospinning, there are many parameters that contribute to the morphology of the fibers. There are three classification categories: (a) solution-related parameters (e.g., solvent conductivity), (b) device-related parameters (e.g., needle-collector distance, applied voltage, speed of the rotating collector), and (c) parameters related to the environment (e.g., humidity) [57]. Each of these parameters can directly affect the morphology of the fibers and fibers can be formed by the method of electrospinning with desired morphology and diameter size by carefully controlling these parameters [57]. Moreover, materials such as metals, ceramics, and glasses can be used as fiber fillers depending on the desired application [58,59,60].

The aim of this study was the preparation of nanocomposite membranes by the incorporation of Ca/Mg/Sr-containing MCM-41 type particles in electrospun copolymer PLGA fibers for the delivery of moxifloxacin and to investigate their structural properties and drug loading/release profiles. 

## 2. Materials and Methods

### 2.1. Synthesis of MSNs

The synthesis of silica-based MSNs, with the nominal composition of akermanite (Ca_2_MgSi_2_O_7_) doped with SrO (40SiO_2_, 40CaO, 18MgO and 2SrO %mol) was performed through a modified sol-gel method, as previously described [28]. Briefly, cetyltrimethylammonium bromide (CTAB) was used as an agent for the mesoporous structure and the final molar ratios were 1TEOS/0.13CTAB/0.4NaOH/1280H_2_O. The white precipitate was washed twice with ethanol and water, dried at 60 °C overnight, and calcinated at 600 °C for 5 h to remove the mesoporous agent. Sodium hydroxide (NaOH, alkaline medium), CTAB, tetraethyl orthosilicate (TEOS), Ca(NO_3_)_2_·4H_2_O, Mg(NO_3_)_2_.6H_2_O, and Sr(NO_3_)_2_ were used as reactants (all reagents from Sigma-Aldrich now Merck KGaA, Darmstadt, Germany)).

### 2.2. Preparation of Solutions for Electrospinning Process

Two different types of PLGA electrospun membranes were fabricated in the present study: a. neat PLGA and b. composite PLGA/MSN membranes. For the preparation of neat PLGA electrospun nanofibers, the electrospinning solutions were prepared by dissolving commercially available PLGA beads (75:25; PLA: PGA) in an organic solvent mixture of chloroform (CHL) and N, N-dimethylformamide (DMF) (50:50% *v*/*v*) in the concentration of 20 wt % (20N group) and 30 wt % (30N group) of PLGA, respectively. For the preparation of composite PLGA/MSN membranes, 10 (20A and 30A group) and 3% *w*/*v* (30B group) of MSNs were added to the electrospinning solution, magnetically stirred overnight at ambient conditions and ultrasonicated for 30 min at room temperature to disperse the MSNs prior to electrospinning. PLGA (PDLG 7507, 75:25 molar ratio, PURASORB^®^) was purchased from Corbion (Amsterdam, The Netherlands).

### 2.3. Electrospinning Process

A standard electrospinning setup (FLUIDNATEK^®^ LE-10, Bioinicia, Valencia, Spain), equipped with a 10 cm diameter rotating drum collector) was used for the fabrication of the electrospun PLGA-based membranes. PLGA-based electrospun membranes were fabricated at a different applied voltage ranging from 18 kV to 22 kV using a high voltage power supply. The ground collection plate of aluminum foil was located at a fixed distance of 8 cm from the needle tip. The speed of the rotated collector was controlled in the range of 400 rpm to 1100 rpm and the feeding rate was set to 600 μL/h (Table 1). The electrospinning process was assessed at room temperature 25.0 ± 0.1 °C. The collected electrospun membranes were dried for 24 h at room temperature and then kept in a vacuum for 3 days.

### 2.4. Characterization

#### 2.4.1. Scanning Electron Microscopy and Energy Dispersive Spectroscopic Analysis (SEM-EDS)

Morphological structures of nano-fiber electrospun PLGA-based membranes were observed using field-emission scanning electron microscopy, JEOL JSM-7610F Plus, supported by an Oxford AZTEC ENERGY ADVANCED X-act energy dispersive X-ray spectroscopy (EDS) system (JEOL Ltd., Tokyo, Japan). A 200 Å thick carbon coating was applied to increase the conductivity of the samples.

The fabricated electrospun membranes for all the above experiments were observed by scanning electron microscopy (SEM) for surface morphology and pore and fiber diameter range, and EDS analysis with beam voltage of 20 kV was carried out in order to qualitatively assess the presence of MSNs in the fibers. The average pore and fiber diameter of the nano-fibered membranes were obtained by measuring the diameter of 100 nanofibers and calculating their average from three SEM micrographs from each sample. The fiber and pore distribution were determined using the following equation (Gauss fitting method):

(1) $$y=y_{o}+\frac{A}{w\sqrt{\frac{\pi }{2}}}e^{-2\frac{{\left(x-x_{c}\right)}^{2}}{w^{2}}}$$

where:

y_o_ = offset
$x_{c}$ = center
***w*** = width
***A*** = area

#### 2.4.2. Attenuated Total Reflectance Spectroscopy (ATR)

Selected fibrous and composite fibrous polymeric membranes were studied using attenuated total reflectance spectroscopy (ATR). The absorption spectra of the freshly prepared membranes were obtained with the use of an ATR Cary 670 spectroscope (Agilent Technologies, Santa Clara, CA, USA), equipped with a diamond attenuated total reflection accessory (ATR), model Gladi ATR (Pike Technologies, Fitchburg, Wisconsin, United States of America), in the mid-infrared range MIR (4–4000 cm^−1^), with a resolution of 4 cm^−1^ and 32 scans.

#### 2.4.3. X-ray Diffraction (XRD)

X-ray diffraction (XRD) patterns were performed with an XRD-diffractometer (Rigaku Miniflex II, Beijing, China) with CuKα radiation for crystalline phase identification (λ = 0.15405 nm for CuKα). The sample was scanned from 5 to 80°, with steps of 0.02°.

#### 2.4.4. Mechanical Properties

Measurements of the tensile mechanical properties of the prepared samples were performed with the use of an Instron 3344 dynamometer (Instron, Norwood, MA, USA), in accordance with ASTM D638, using a crosshead speed of 10 mm/min. Dumbbell-shaped tensile test specimens (central portions 0.20 ± 0.01 mm thick, 20 mm gauge length) were cut from the samples in a Wallace cutting press. From the stress-strain curves, Young’s modulus and tensile strength were determined. Ten specimens were tested from each sample.

#### 2.4.5. Equilibrium Swelling Performance and In Vitro Degradation

The swelling capacity was carried out using phosphate-buffered saline (PBS pH 7.4). Each sample was weighed before immersion (***W*_1_**) and immersed for various time intervals (0, 1, 4, 7, 10, 14, 28 days). The solution on the surface of the samples was removed using filter paper and then weighed (***W*_2_**). Absorbed water/solution ratio was then calculated using the equation:

(2) $$\mathrm{Swelling}\;\mathrm{Capacity}=\frac{W_{2}-W_{1}}{W_{2}}\;\times \;100\%$$

For the degradation test of each of the selected membranes, 10 × 60 × 0.2 mm sheets of membrane material were prepared. Each specimen was placed in 10 mL 0.1 M phosphate-buffered saline (PBS, pH 7.4). The specimens were stored in an incubator (MaxQ 4400 incubator) at 100 rpm, 37.0 ± 0.5 °C for 7, 14 και 28 days [61,62,63]. The PBS solution was replaced weekly. All specimens were washed with distilled water to remove residual solution and the wet weight of each specimen was measured (Kern ABS). To measure the dry weight, specimens were dried in a vacuum oven for 48 h at 40 ± 2 °C. Following drying, each specimen was weighed at 2 h intervals until recorded mass changes were under 0.1%. The difference between the initial mass of the membrane (***W_o_***) and the mass after the immersion (***W_t_***) provided the initial mass of the degraded sample, thus the (%) weight loss of the sample was derived using the following equation:

(3) $$\mathrm{Mass}\;\mathrm{Loss}=\frac{W_{o}-W_{t}}{W_{o}}\;\times \;100\%$$

#### 2.4.6. Drug Loading and Drug Content Quantification

Neat PLGA membranes (30N2DL): In the case of neat PLGA membranes (30N2), 1 g of the MOX was directly mixed into the electrospinning solution (10 mg/mL) prior to electrospinning; the electrospinning conditions were the same as described in part 2.3 for the sample 30N2.

Composite PLGA/MSN membranes (30A2DL): In the case of composite membranes, 1 g of MSNs was dispersed in 100 mL of moxifloxacin hydrochloride (MOX) methanol solution (10 mg/mL), vigorously stirred for 24 h at 37 °C, and then dried in air for 24 h (“MOX-loaded MSNs”, hereafter). The MOX-loaded MSNs were added to the electrospinning solution (as described in 2.2 for the 30A2 sample).

After the electrospinning process, pre-weighted samples were cut from 30N2DL and 30A2DL samples and dissolved in 20 mL of a mixture of DCM/MeOH (50:50 *v*/*v*) and the resultant solution was analyzed for MOX content using high performance liquid chromatography (HPLC) technique. The column used was a CNW Technologies Athena C18, 120 A, 5 μm, 250 mm × 4.6 mm at a column temperature of 25 °C. The mobile phase consisted of acetonitrile and ultra-pure water ACN/H_2_O (acidified with phosphoric acid at final pH = 5) 40/60 *v*/*v*, at a flow rate of 1.0 mL/min). Concentration determination was performed using an HPLC-UV apparatus at 278 nm and was based on a previously created calibration curve. The injection volume was 20 μL. The calibration curve was created by diluting a stock methanol solution of 40 ppm MOX to concentrations of 0.025, 0.05, 0.1, 0.5, 1, 2.5, 5, 10, 20, 30, 40 and 50 ppm using mobile phase.

Drug loading (DL) was calculated according to the following equation:

(4) $$\mathrm{DL}=\;\frac{W_{drug\;in\;the\;membranes}}{W_{drug}}\;\times \;100\%$$

#### 2.4.7. In Vitro Drug Release

For the in vitro release studies, DISTEK Dissolution Apparatus Evolution 4300, equipped with an autosampler using the paddle method (USP II method), was applied. A film was placed on each dissolution vessel corresponding to approximately 50 mg of each formulation in an appropriate transdermal patch holder, with its application side up. The test was performed at 37 ± 1 °C with a rotation speed of 50 rpm. The dissolution medium was 500 mL of a phosphate-buffered saline (PBS), pH = 7.0. Two (2) mL of the aqueous solution was withdrawn from the release media and analyzed for MOX content with the aid of a HPLC technique using the same conditions as described in 2.4.6.

#### 2.4.8. Hemocompatibility Assay

The hemocompatibility of erythrocytes with the electrospun membranes (EMs) and/or un/loaded mesoporous nanoparticles (concentration) was evaluated under the following conditions: Diluted erythrocytes (DEs) (5% hematocrit) were exposed with different masses (2, 1, and 0.5 mg) of EMs. The above mixtures were then incubated (Thermomixer-Biosan) at 37 °C for 60 min and 24 h. The supernatant of untreated erythrocytes was used as the negative control (Ctrl-) and erythrocytes treated with lysis buffer were used as the positive control. All the samples were further centrifuged at 2000 rpm for 1 min and a microplate reader was used to measure the absorbance of hemoglobin release in the supernatant of treated samples. The absorbance value of hemoglobin at 541 nm was measured with the reference wavelength of 700 nm. The percent of hemolysis was calculated as follows: hemolysis % = [(sample absorbance − negative control) (positive control − negative control)] × 100% (1). Statistical analysis was performed using a paired sample *t*-test. The level of statistical significance was set at 0.05. Whole blood in EDTA tubes was drawn from blood donors of the General Hospital of Naousa, Greece. The confidentiality of the participants was wholly preserved. The ethical committee of the hospital (ID_233205920) approved the study following their good clinical practice guidelines and the Declaration of Helsinki.

## 3. Results

### 3.1. Scanning Electron Microscopy and Energy Dispersive Spectroscopic Analysis (SEM-EDS)

**Figure 1:**
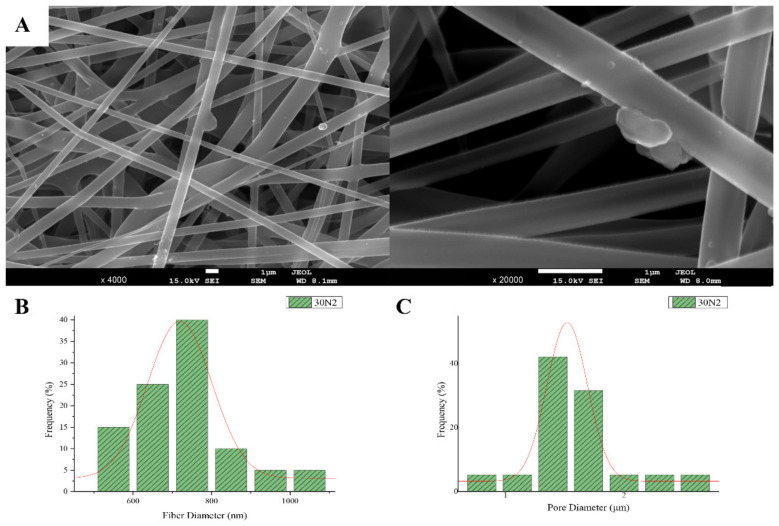
SEM microphotographs at ×4000 and ×20,000 magnification (**A**), and fiber (**B**), and pore (**C**) diameter distribution of sample 30N2 (neat PLGA membranes). The average fiber diameter of the neat PLGA membranes (30N2) was 720 nm, while the average pore diameter was approximately 1.5 μm. Τhe addition of MSNs led to a significant reduction in fiber and pore diameter (Figure 2).

**Figure 2:**
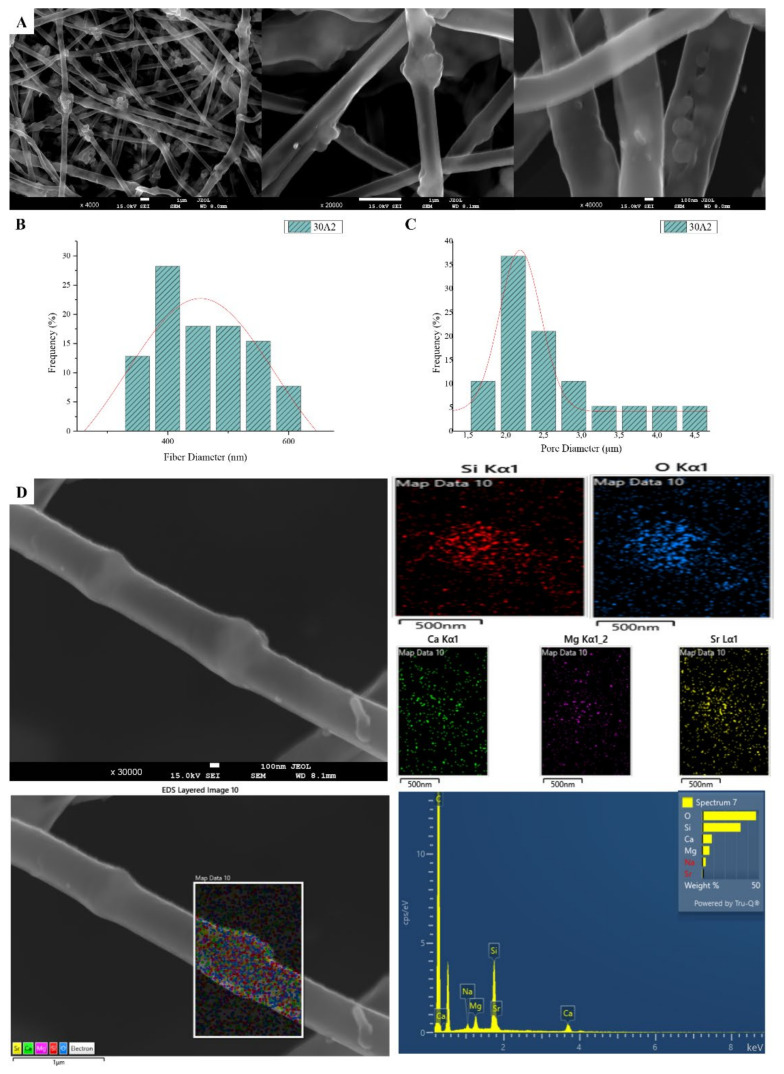
SEM microphotographs at ×4000, ×20,000 and ×40,000 magnification (**A**), fiber (**B**) and pore (**C**) diameter distribution and EDS analysis (**D**) performed at 20 kV of the sample 30A2 (composite PLGA/MSN membranes loaded with 10% *w*/*v* MSNs).

**Figure 3:**
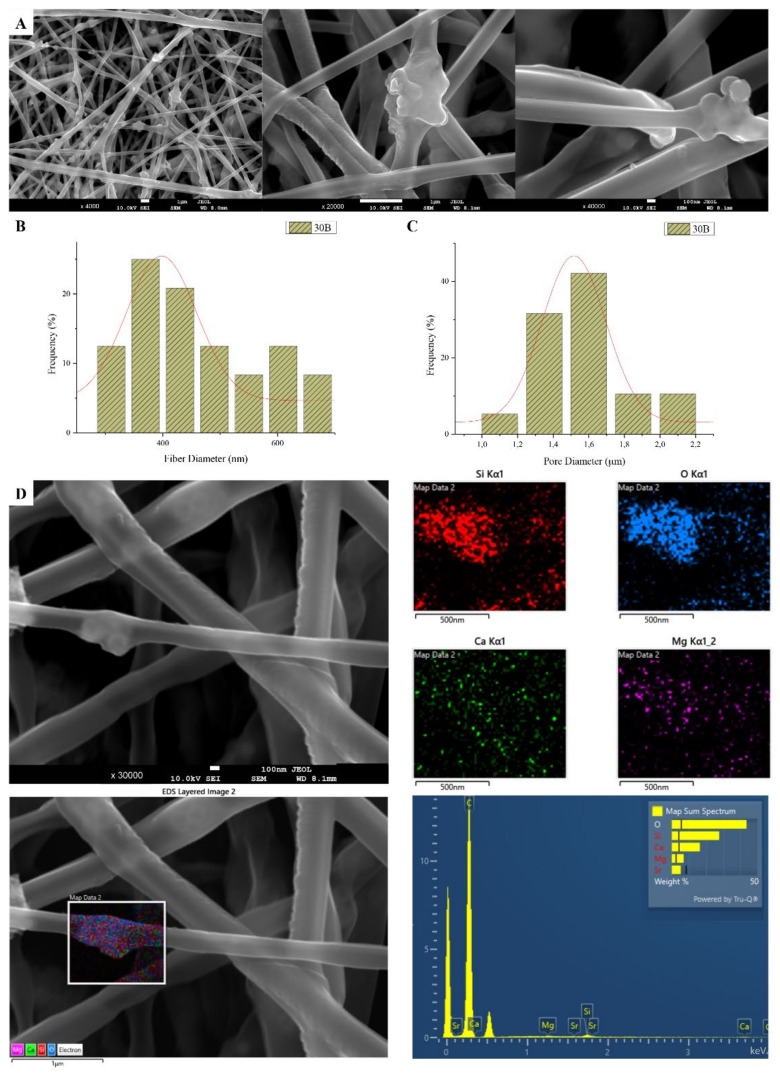
SEM microphotographs at ×4000, ×20,000 and ×40,000 magnification (**A**), fiber (**B**), and pore (**C**) diameter distribution, and EDS analysis (**D**) performed at 20 kV of the sample 30B (composite PLGA/MSN membranes loaded with 3% *w*/*v* MSNs) 30B.

The sample names and the average diameter of the fibers and pores of all the obtained electrospun membranes, as measured from the SEM microphotographs, are given in Table 1. The samples (30N2, 30A2 and 30B) that were fabricated using 22 kV applied voltage, 8 cm distance from the needle tip, at 800 rpm speed of the rotated collector, and 600 μL/h feeding rate, were the optimal ones. Figure 1, Figure 2 and Figure 3 show the SEM microphotographs and the average fiber and pore diameter of the optimal samples consisting of 30% and 20% *w*/*v* PLGA with the addition of 0 (Figure 1), 3 (Figure 3) or 10% *w*/*v* (Figure 2) MSNs. Figure 2 and Figure 3 also show the EDS results, which confirm the presence of MSNs (presence of silicon, calcium, magnesium, calcium, and strontium ions).

SEM images of groups 30A and 20A at ×4000 magnification (data not shown) showed better dispersion of the added nanoparticles within the fibers in the sample 30A2 (Figure 2), in combination with increased porosity and larger fiber diameter. Consequently, the sample 30N2 (0% MNSs) was used as control and samples 30A2 (10% MSNs), and 30B (addition of 3% *w*/*v* MSNs), obtained under similar synthesis conditions, were selected for further study.

The measurements of all fibrous scaffolds revealed that increase in the voltage led to an increase in the size of the fibers and the size of the pores. In addition, increasing the speed of the rotating collector did not seem to significantly affect the average fiber size, but it did seem to result in a small increase in pore size. However, there was a simultaneous increase in fiber diameter with increasing pore size.

### 3.2. Attenuated Total Reflectance Spectroscopy (ATR)

**Figure 4:**
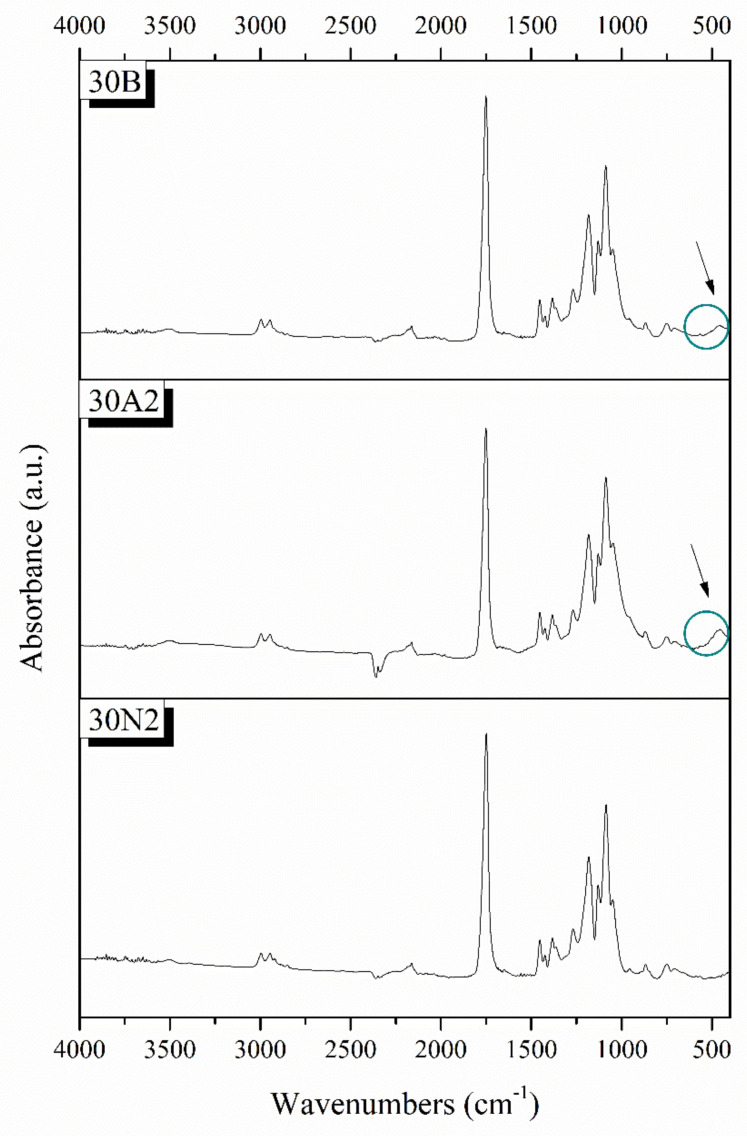
Chemical characterization PLGA-based membranes by ATR spectra. 30N2: neat PLGA membranes, 30A2: composite PLGA/MSN membranes loaded with 10% *w*/*v* MSNs, 30B: composite PLGA/MSN membranes loaded with 10% *w*/*v* MSNs.

Typical spectra of PLGA polymer-based and composite membranes were found from ATR analysis for the selected samples 30N2, 30A2, and 30B (Figure 4). The bands at 1751 and 1088 cm^−1^ were attributed to the vibrations of carbonyl and C-O-C stretching peaks of the PLGA group. The peaks around 1455 and 1423 cm^−1^ were attributed to the C-H stretching in methyl groups. These particular peaks were assigned to the C-CH_3_ stretching vibrations [64]. The peak at 1184 cm^−1^ was attributed to the vibration of the C-O-C bond, the peak around 1049 cm^−1^ was also attributed to the C-CH_3_ vibration, while the peak at 1270 cm^−1^ was attributed to the PLGA ester groups [65]. The spectra of the composite membranes 30A2 and 30B showed a peak around 460 cm^−1^, which was not observed in the spectrum of sample 30N2 (neat PLGA electrospun membrane). This peak was attributed to the bending vibration of the Si-O-Si bond due to the presence of silica-based mesoporous nanocarriers. In addition, the presence of mesoporous nanocarriers was also confirmed by the widening of the broad peak between 900 and 1542 cm^−1^, because the wide peak of typical silicate glasses (900–1200 cm^−1^ was attributed to the asymmetric vibration of the Si-O-Si bond) overlaps with those of the synthetic polymer PLGA in this region [28].

### 3.3. Mechanical Characterization

**Figure 5:**
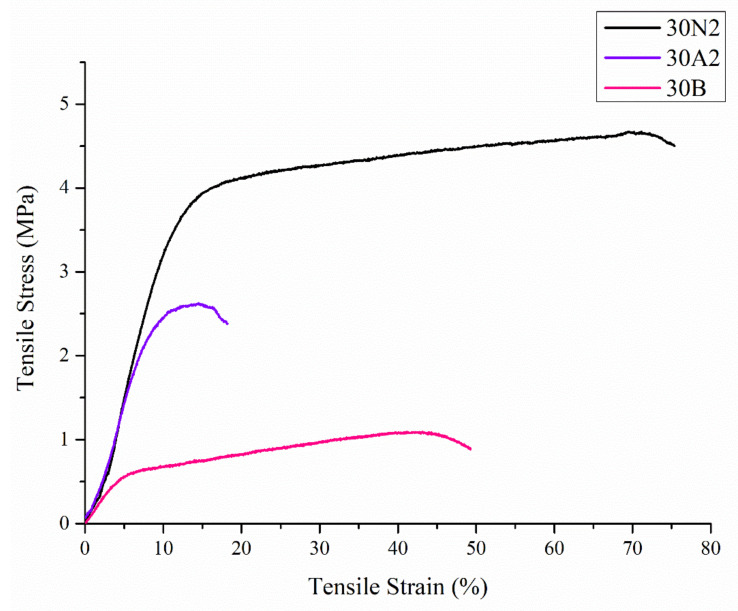
Representative stress-strain curves of electrospun membranes. 30N2: neat PLGA membranes, 30A2: composite PLGA/MSN membranes loaded with 10% *w*/*v* MSNs, 30B: composite PLGA/MSN membranes loaded with 10% *w*/*v* MSNs.

Uniaxial tensile tests were performed on the electrospun membranes. Figure 5 shows representative stress-strain curves of each sample. In Table 2, the values related to ultimate tensile strength (UTS) and tensile strain are reported.

Compared with the neat PLGA membranes (30N2), for the PLGA/MSN membranes (30A2 and 30B) the breaking strength decreased with the addition of MSNs. This may be due to poor interfacial adhesion between MSNs and PLGA or poor distribution of the nanoparticles in the polymeric matrix.

### 3.4. In Vitro Degradation and Equilibrium Swelling Performance

**Figure 6:**
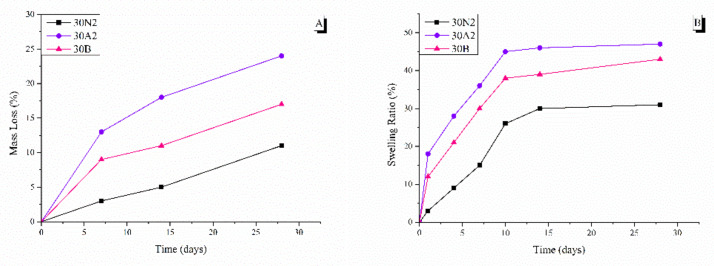
Mass loss (**A**) and swelling ratio (**B**) at different time points. 30N2: neat PLGA membranes, 30A2: composite PLGA/MSN membranes loaded with 10% *w*/*v* MSNs, 30B: composite PLGA/MSN membranes loaded with 10% *w*/*v* MSNs.

The degradation rate of neat PLGA and composite membranes was performed for 0, 7, 14, and 28 days, as shown in Figure 6. It was found that the weight loss of all groups increased over time. More specifically, composite membranes (30A2 and 30B) showed higher weight loss values than the neat PLGA membranes (30N2) group. In addition, increase in the amount of MSNs increased the weight loss. However, the hydrophilicity of the mesoporous nanocarriers containing samples (30A2 and 30B) was increased, as shown in the swelling results (Figure 6). More specifically, composite membranes showed higher swelling than neat PLGA membranes, which might be due to improvement in the hydrophilicity of PLGA membranes after the addition of MSNs [66].

### 3.5. Drug Loading and Release

The drug loading of MOX for the electrospun nanofibers was also studied. Table 3 summarizes the results of the loading of moxifloxacin for each sample. The loading percentages of the 30N2DL and 30A2DL samples were 37.2 and 27.5%, respectively, indicating an average loading capacity.

**Figure 7:**
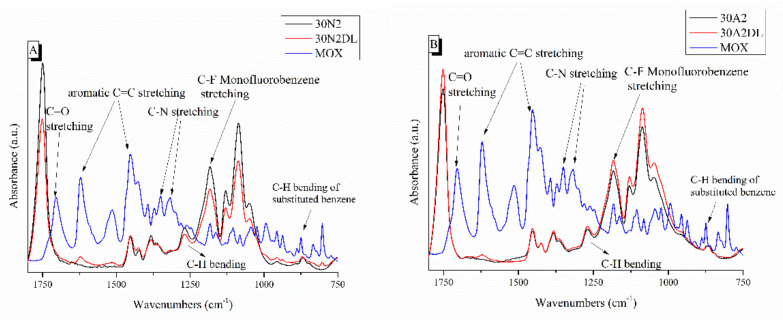
ATR spectra of PLGA-based membranes before and after loading with moxifloxacin. 30N2, 30N2DL, and MOX (**A**) and 30A2, 30A2DL, and MOX (**B**). 30N2: neat PLGA membranes, 30N2DL: drug-loaded neat PLGA membranes, 30A2: composite PLGA/MSN membranes, 30A2DL: composite PLGA/MOX-loaded MSNs membranes.

Figure 7 shows the ATR spectra of fibrous membranes before and after drug encapsulation together with the moxifloxacin spectrum as a reference. The presence of the characteristic peaks corresponding to moxifloxacin in the spectra of the encapsulated fibrous membranes confirmed the presence of the drug in the samples, while the small displacement of the MOX peaks indicated the interaction of the polymer with the drug. The peaks presented in the moxifloxacin spectra around 1415 to 1475 cm^−1^, 2521 cm^−1^ and 1355 cm^−1^ were attributed to the vibrations of ((CH)-CH_2_), ν(NH_2_^+^) and δ_b_(-CH_2_), respectively [67]. In addition, the peak at 1621 cm^−1^ was attributed to the bending vibration of the N-H bond (due to presence of quinolones), the peaks at 801 and 990 cm^−1^ were attributed to the bending vibration of the C-H bond and the peak around 1711 cm^−1^ corresponded to the vibration of the deformed δ_b_(COO-) bond [28,30,67,68,69,70]. A more limited presence of certain moxifloxacin peaks was observed in the MSNs incorporated sample (30A2DL) spectra, indicating a lower drug encapsulation than the neat PLGA sample (30N2DL). This was expected due to the limited amount of the incorporated MSNs (10% *w*/*v*) and the low percentage of encapsulation of the drug in them (~31%). In addition, this was also confirmed by the determination of the loading of the MSN-incorporated fibrous membranes (30A2DL) (27.5 ± 0.2%), which was lower than the fibrous membranes without MSNs (30N2DL) (37.2 ± 0.1%) sample.

**Figure 8:**
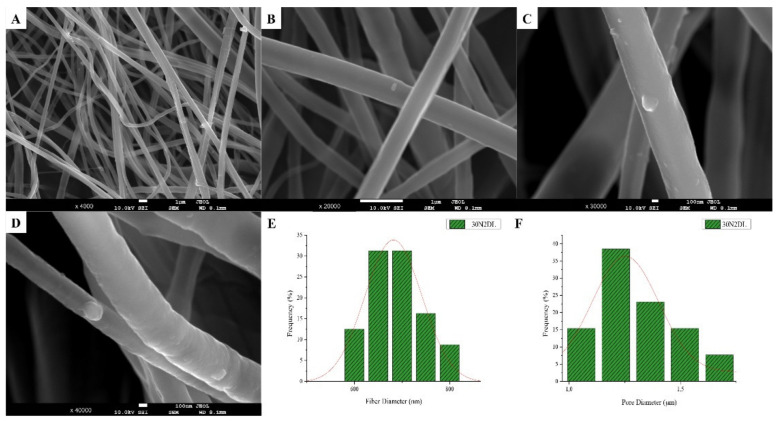
SEM microphotographs (**A**–**D**) and fiber (**E**) and pore (**F**) diameter distribution of sample 30N2 after drug loading (30N2DL).

**Figure 9:**
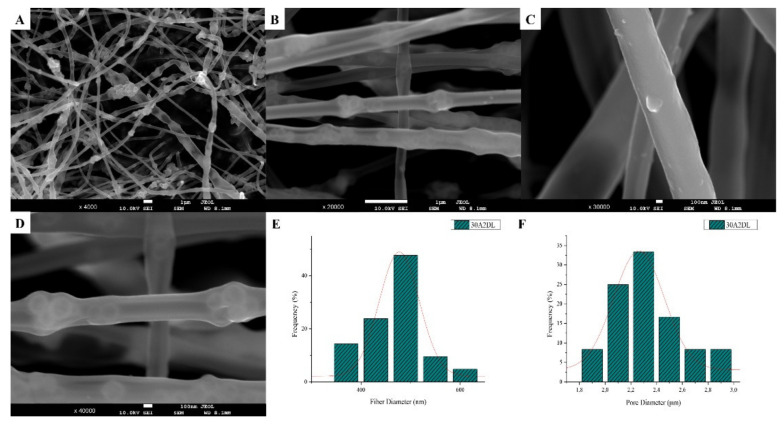
SEM microphotographs (**A**–**D**) and fiber (**E**) and pore (**F**) diameter distribution of composite PLGA/MOX-loaded MSNs (sample 30A2DL).

The SEM microphotographs and the mean size distribution of the fibers and pores of the fabricated membranes after the encapsulation of the drug are presented in Figure 8 and Figure 9. It can be observed that the encapsulation of the drug inside the fibers for the 30N2DL sample resulted in a small reduction in the fiber cross-sections (by regions) that are necessary for the creation of pores and their interconnection. However, it seems that there were no significant changes in the morphology of the fibers or in the size distribution of their diameters and pores (~700 nm and 1.4 μm). SEM micrographs of the 30A2DL sample confirm the very good dispersion of mesoporous nanoparticles within the fibers, even after the encapsulation of the drug in the mesopores. This fact is confirmed by the average size distributions of the diameter of the fibers and pores that do not seem to have been affected by the encapsulation of the drug (~430 nm and 4 μm respectively).

**Figure 10:**
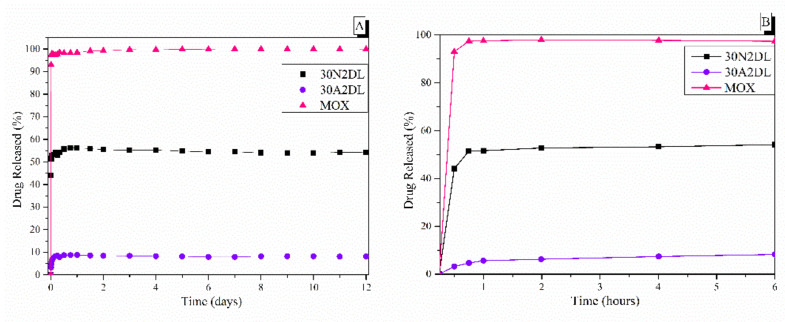
The in vitro release rate of moxifloxacin from fibrous and composite fibrous electrospun membranes at pH 7.4 after 12 days (**A**) and after 6 h (**B**) of immersion in PBS. 30N2: neat PLGA membranes, 30A2: composite PLGA/MOX-loaded MSNs membranes.

PLGA copolymer undergoes degradation by hydrolysis or biodegradation through cleavage of its backbone ester linkages into oligomers and, finally, monomers. The degradation process for these polymers mainly takes place over the entire polymer matrix, leading to a uniform mode of erosion, called a ‘bulk’ pathway [71]. In this case, the water penetration into the matrix is higher than the rate of polymer degradation. The degradation of PLGA copolymer is dependent on many factors, such as bulk diffusion, surface diffusion, bulk erosion and surface erosion. As a result, the release rate pattern is often unpredictable. Figure 10 shows the dissolution profiles of pure moxifloxacin hydrochloride and prepared PLGA/MOX and PLGA-MSN/MOX electrospun nanofibers in the PBS medium.

Figure 10 also shows the biphasic drug release rate of MOX from the fibrous scaffolds loaded with drug either directly into the fibers (30N2DL) or into the MSNs within the fibers (30A2DL). Pure moxifloxacin was released rapidly, as expected, and reached a plateau (stabilization) of 98% in less than 24 h. A different pattern of drug release as a result of PLGA biodegradation was observed (Figure 10), depending on whether the drug was loaded on the PLGA scaffold (30N2DL) or in the MSN-loaded PLGA scaffold (30A2DL). In the first case, a biphasic release was observed. The initial burst release in the first hours can be attributed to the drug released from the surface, in direct contact with the medium, as a function of solubility as well as penetration of water into the polymer matrix. Matrices having higher drug content possess a larger initial burst release than those having lower content because of their smaller polymer to drug ratio. At a second stage, the drug is progressively released through the underlying thicker drug depleted layer. This creates a passage for the drug to be delivered by diffusion and erosion until complete polymer solubilization. Drug type also plays an important role in attracting the aqueous phase into the matrix. The initial burst was followed by a slower and more controlled release stage, and after that, a very slow release, reaching almost a plateau, was recorded after 6 h, while almost 52% of the MOX was released after 12 days. In the second case (30A2DL), when MOX was adsorbed onto the MSNs dispersed in the PLGA matrix, a different dissolution pattern was observed. MOX release achieved its maximum, reaching a plateau in less than 24 h, while ~8% of MOX was released after 12 days of study. According to our previous findings, the release rate of the drug directly from the MSNs showed a prolonged release profile showing a plateau after 2 days, releasing approximately 45% of the MOX. This limited and prolonged release was in part attributed to the strong interactions between the MSNs Si-OH groups and the drug and the low percentage of loaded MSNs, but mostly to the gradual and slow degradation of the polymer that restricted the exposure of MSNs and consequently the release of the drug [28]. 

**Figure 11:**
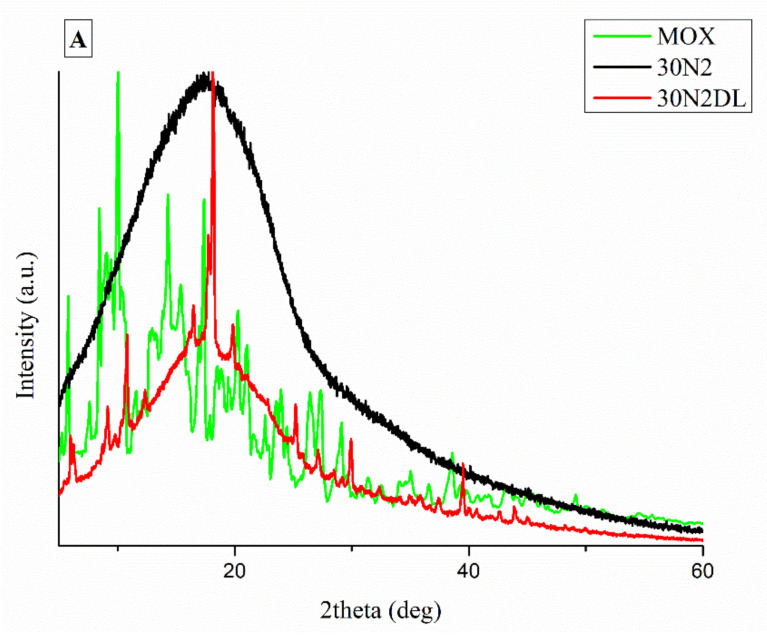

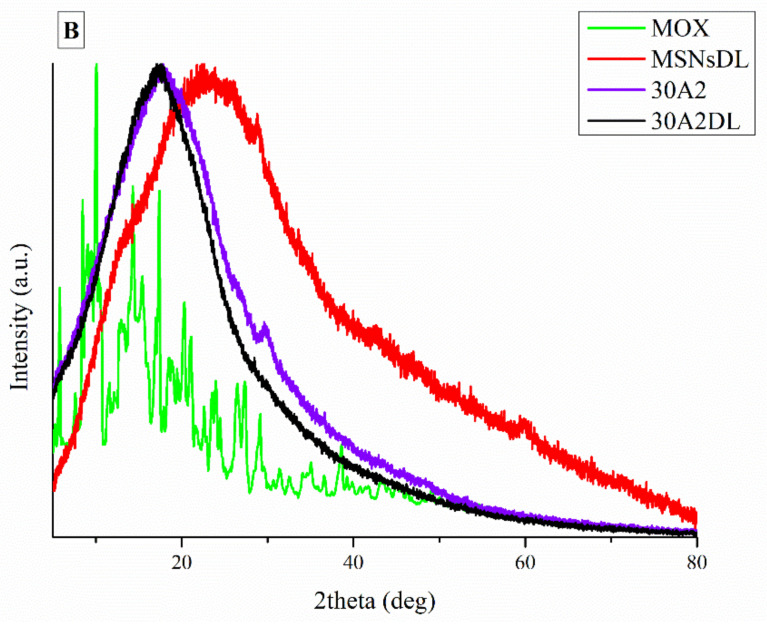
XRD patterns of neat PLGA membranes before (30N2) and after MOX-loading (30N2DL) compared to pure MOX (**A**), and XRD patterns of composite membranes before (30A2) and after loading with MOX-loaded MSNs (30A2DL) and MOX-loaded MSNs compared to pure MOX (**B**).

It is well known that the crystallinity of the pharmaceutical ingredient affects the dissolution rate and bioavailability. For this reason, XRD studies were performed to examine the crystallinity of MOX before and after its adsorption onto MSNs and the electrospun membrane. Figure 11A shows the respective patterns of the neat membranes (30N2) which are amorphous in the region between 5 and 60° and of MOX, which is highly crystalline, showing its distinct peaks at 5.96, 8.66, 10.28, 14.63 and 17.56° at 2θ. We observed that in loaded PLGA polymer some lower intensity peaks, close to the moxifloxacin peaks, were recorded. These peaks may be attributed to the different crystal form of moxifloxacin compared to the neat drug [72]. The absence of distinct diffraction peaks, after the adsorption of MOX in MSNs (MSNsDL) shown in Figure 11B, suggests amorphization of the drug in the mesoporous carrier. This amorphization could be due to the high dispersion of MOX on the mesopore surface of MSNs or to interactions taking place between silanol groups of MSNs and MOX [73].

**Figure 12:**
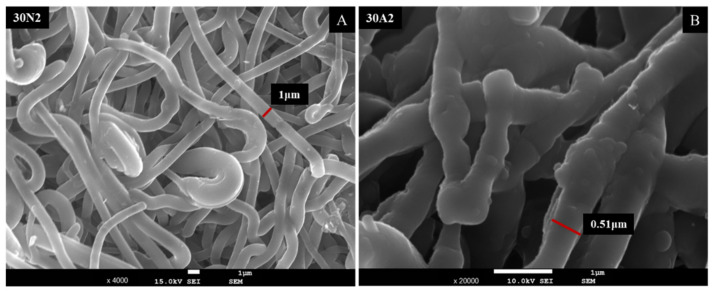
SEM micrographs of samples 30N2 (**A**) and 30A2 (**B**) after 12 days of drug release in PBS solution at 37 °C. 30N2: drug-loaded neat PLGA membranes, 30A2.: composite PLGA/MOX-loaded MSNs membranes.

Figure 12 shows the SEM micrographs after the drug release into PBS medium. As shown in the pictures of the two samples, the membrane fibers were clearly larger than they were before the drug was released. More specifically, the size of the fiber diameter for the neat PLGA membranes (30N2DL), as previously analyzed, was about 700 nm, while after drug release, it was about 1 μm. For the composite membranes (30A2DL), the fiber size was initially about 430 nm, while after drug release it was about 510 nm. The increase in fiber diameter was due to the swelling of the fibers within the PBS medium. 

### 3.6. Hemocompatibility Assay

**Figure 13:**
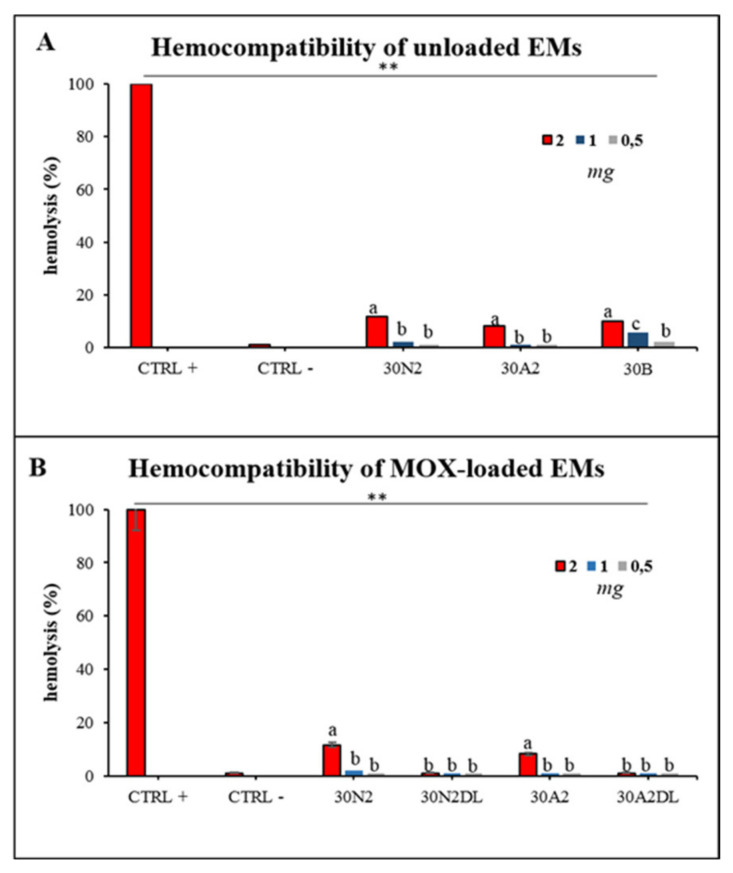
Hemolytic capacity of the electrospun membranes before (**A**) and after (**B**) the encapsulation of moxifloxacin. ** Indicates statistically significant difference (*p* < 0.001) between CTRL + and Ems-treated erythrocytes, while different letters suggest statistically significant differences (*p* < 0.05) among concentrations. 30N2: neat PLGA membranes, 30N2DL: drug-loaded neat PLGA membranes, 30A2: composite PLGA/MSN membranes, 30A2DL: composite PLGA/MOX-loaded MSNs membranes.

Hemocompatibility assay of electrospun membranes after direct exposure to healthy human erythrocytes was performed after 60 min and 24 h of incubation. Figure 13 presents the hemolytic activity of the newly synthesized electrospun membranes at body temperature (37 °C) after 24 h of exposure. The tested biomaterials did not induce hemolysis 60 min after direct contact with the healthy erythrocytes. Hemolysis appeared only at 24 h of incubation for 2 mg of samples for all the unloaded membranes tested (Figure 13A). The hemocompatible mass for all the tested membranes was found to be less than 1 mg. Moreover, loading with MOX appeared to be protective for erythrocytes, indicating the positive role of drug loading in electrospun membranes.

## 4. Discussion

In this study, nanocomposite membranes were fabricated by the incorporation of Ca/Mg/Sr-containing MCM-41 mesoporous nanoparticles in electrospun copolymer poly(lactic-glycolic acid) (PLGA) fibers for the delivery of the antibacterial drug moxifloxacin, with optimum morphology, drug release, degradation rate and hemocompatibility. According to our previous study [74], the selected mesoporous nanocarriers presented good hemolytic behavior and cell proliferation while maintaining adequate moxifloxacin loading and having a sustained release rate [28]. The MSNs also provided high surface area (700 m^2^/g) and pore volume (1.618 cm^3^/g) and typical mesoporous structure, making them ideal drug carriers for drug loading and release studies. These MSNs have shown crystalline hydroxyapatite formation after immersion in simulated body fluid [28] and their incorporation in electrospun PLGA membranes can provide an additional benefit towards bone healing and regeneration [75]. Electrospinning has gained much attention in the last decade as an effective means of producing nano- to micro-scale polymer fibers that mimic the natural extracellular matrix. The high porosity, the interconnection of the pores, and the ratio of high surface to the total volume of the fibrous scaffolds make them particularly favorable for cell adhesion and growth [76]. Cellular adhesion is essential for a number of applications of tissue engineering. Pore size, porosity, and pore connectivity determine cell adhesion and tissue growth in the fibrous scaffold and affect several cellular processes [74,77,78,79,80,81]. 

According to previous studies, it is possible to achieve a correspondence between the size of the pores and the size of the fiber diameter [76]. More specifically, larger pores can be created by increasing the diameter of the fibers. This is an approach described by a number of research groups as a way of increasing the pore size of fibrous scaffolds obtained by the technique of electrospinning (using synthetic or natural polymers) [82,83,84,85,86]. Statistical modeling predicts a relationship between fiber diameter and the pore size of fibrous scaffolds, where a larger fiber diameter is associated with an increase in pore size [87]. This is consistent with experimental data obtained for a number of polymers, including PLGA [86]. Moreover, according to Motamedi et al., there is a correlation between collector speed and pore diameter. More specifically, they observed that increasing the speed of the collector leads to increasing the diameter of the fibers [88]. This is in line with the findings of the present study. More specifically, it was found that increase in the speed of the rotating collector led to an increase in fiber diameter and a small increase in the pore size. The study of the parameters for the successful fabrication of the electrospun membranes was conducted using different polymer concentrations and MSNs percentages in the solution and different voltage and collector rotating speed values. SEM micrographs revealed the formation of smaller fibers as the polymer concentration decreased, while increase in the voltage led to an increase in the fiber diameter and the size of the pores. According to the literature, several studies on the effect of applied voltage on the size of the fiber diameter are contradictory. More specifically, as the applied voltage is a critical factor for the electrospinning technique, it must be higher than the threshold voltage value so it can cause charged jets to be ejected from the Taylor cone [51,89,90]. When the applied voltage value is not high enough, the polymer extraction (at the end of the needle) is suspended. However, as the voltage value increases, it is possible to create a jet that will be extracted from the Taylor cone. In that case, it is possible to fabricate fibers without beads. The effect of the applied voltage on the fiber diameter is also a controversial issue [51,91]. It is mainly reported that higher voltages facilitate the formation of large diameter fibers [51,92]. However, it has been reported that the higher voltage may increase the electrostatic repulsive force on the charged jet, favoring a reduction in the fiber diameter [91]. It has also been shown that the higher the voltage, the greater the chance of bead formation. Thus, we can conclude that the voltage affects the diameter of the fibers, but the mode of effect varies depending on the concentration of the polymer solution and the distance between the tip and the collector [93].

SEM images of groups 30A and 20A indicated a better dispersion of the intermediate nanopores within the fibers in the sample 30A2, in combination with the increased porosity and the large diameter of the fibers. The samples 30A2 (addition of 10% *w*/*v* MSNs) in combination with neat membranes 30N2 and 30B (addition of 3% *w*/*v* MSNs) were obtained under similar synthesis conditions and were selected for further study. The study of the mechanical properties of the membranes revealed that incorporation of MSNs in PLGA fibers led to a reduction in the breaking strength. Similarly, according to Jia et al. [69], who assessed the effects of andrographolide-loaded MSNs incorporation in poly(lactic-co-glycolic acid) (PLGA) electrospun nanofibers in wound-healing applications, it was found that the Young’s modulus of the composite membranes decreased as the percentage of MNSs increased due to the poor interfacial adhesion between the incorporated drug-loaded-MSNs and PLGA. This finding is further supported by the presence of agglomerates in the synthesized MSNs. It is well known that the rate of agglomeration increases with increase in nanofiller percentage and decrease in the particle size [94]. The aggregation of nanoparticles can be observed when the particles are loosely bonded together and can be easily separated when mechanical forces are exerted. The presence of the agglomeration has been attributed to the direct mutual attraction between the nanoparticles by Van der Waals forces or chemical bonds. In addition, the presence of agglomeration reduces the efficiency of incorporating the nanoparticles into the polymer matrix, which ultimately leads to a reduction in the mechanical properties of the samples, as was observed in the present study [94]. In the present study none of the proposed strategies to avoid agglomeration was carried out. One such strategy would be the use of a surfactant and charged agent to separate particles by repulsive electrostatic forces and to integrate them into the polymeric matrix, or the use of a silane agent that could provide a better bond with the polymer, improving the mechanical properties of the membranes in terms of tensile strength and Young’s modulus to ensure integrity and allow easier handling and placement, and also the encapsulation and release profile of active substances [94,95]. 

The addition of mesoporous nanocarriers to the membrane fibers led to an increase in both weight loss and swelling. This is in agreement with Jia et al. who also studied the effect of mesoporous nanocarriers on the biodegradation rate and swelling of fibrous scaffolds based on the synthetic polymer PLGA for 14 days [66]. According to their findings, the weight loss of all groups increased over time. The PLGA/MSN groups showed higher weight loss than the neat PLGA group. As the percentage of added mesoporous nanocarriers increased, weight loss also increased. However, due to the increased hydrophilicity of PLGA/MSN composite fibrous scaffolds, an increase in the swelling ratio was observed. This finding was also confirmed by Li et al. In their study, they incorporated the bioglass 45S5 into the synthetic polymer PLGA [58]. The bioglass was found to accelerate mass loss in the first eight weeks. In addition, Jia et al. found that PLGA/MSN composite scaffolds showed higher swelling than neat PLGA fibrous scaffolds [66]. They also observed that the swelling ratio increased with increase in the percentage of added intermediate nanoparticles, due to the improvement in hydrophilicity with the addition of partial nanoparticles. So, there is a relationship between hydrophilicity, degradation and swelling. Increase in the amount of incorporated MSNs can lead to an increase in hydrophilicity which can affect degradation rate and swelling by increasing both. Electrospun membranes designed to be used in GBR should retain their integrity for at least 4–6 weeks to prevent soft tissue downgrowth and allow optimized periodontal tissue regeneration [6,96]. In the present study, although MSNs resulted in a higher degradation rate, membranes still retained their structure after 28 days, presenting a mass loss less than 25%, rendering them applicable in clinical practice. As shown in the literature, different blends of natural and/or synthetic polymers have been developed for the fabrication of electrospun membranes for GBR, such as PLA/PLGA, poly-d,l-lactic acid (PDLLA)/PLGA, hydroxyapatite/chitosan/gelatin, PCL/gelatin etc [97]. Different polycaprolactone (PCL)/gelatin electrospun membranes presented tailored biodegradability, to meet the in vivo rate of bone regeneration, and presented a mass loss of 50% after three months [98]. By altering PLGA percentage and the quantity of loaded MSNs, monitoring of the degradation rate can be further optimized to meet clinical needs.

In our study, composite membranes showed a significantly more controlled release rate of moxifloxacin than neat PLGA membranes, which can be attributed to the structure of the composite fibrous membranes. Fibrous scaffolds loaded with drugs without the use of a nano-carrier, which have been fabricated by the electrospinning technique, have been used as releasing agents for various types of drugs, wherein the drug delivery profiles can be controlled by modifying fabrication conditions [59]. The release of the drug from PLGA fibers occurs by direct diffusion of the drug from the fibers into the release medium, as occurred for sample 30N2DL. In particular, water molecules were diffused through the fiber nanopores and then the MOX was rapidly diffused from the nanopore wall into the buffer. As the water molecules diffused into the composite fibers through the nanopores the interaction between the polymer chains was reduced, so the chains were loosened and the fibers swelled [60]. However, in the 30A2DL composite membranes, the drug was trapped in the mesopores of the incorporated MSNs in the PLGA fibers. The drug was first released by diffusion from the MSNs into the PLGA fibers and then diffusion from the fibers into the medium. This sequential diffusion of the drug through two layers (MSNs and PLGA fibers) initially led to a low burst release and prolonged release of the drug from the fibers of composite membranes [59]. Thus, such an approach could potentially be applied as a strategy to achieve sustained release of various types of drugs and/or active substances. Hu et al. studied the loading and release rate of ibuprofen directly from synthetic PLLA fibrous membranes and from modified MSNs dispersed in PLLA fibers, fabricated by the electrospinning technique. They observed that PLLA-MSN-ibuprofen fibrous composite membranes showed significantly lower initial release of the active substance (6% release in the first 12 h) compared to the release rate of PLLA-IBU fibrous membranes (46% release in the first 12 h) in in vitro tests [59]. Similarly, Song et al. studied the dual release system of the active substances fluorescein (inside the fibers) and rhodamine B (loaded in MSNs) from the PLGA/MSNs composite fibrous electrospun membranes. They observed that the release of both drugs was independent and that there were no significant interactions between the two drugs during release. Then, they observed that in all membranes, fluorescein was rapidly released in the first 12 h at a rate of 50–60%. The release rate was then slowed to show a sustained release (approximately 90% of the total fluorescein amount after 324 h) [60]. The release of rhodamine B from the PLGA/fluorescein/MSNs/rhodamine B composite membranes from the fibers was prolonged, with only ~37% of the loaded rhodamine B released after 324 h. This confirmed the contribution of rhodamine B encapsulation in the intermediate nanoparticles, which enriched the PLGA polymeric matrix, to its prolonged release. In summary, the use of mesoporous nanoparticles as carriers of active substances is an efficient method for the prolonged/sustained release of drugs by fibrous scaffolds for applications of GTR. By tailoring the quantity of MSNs, their loading capacity, their bonding mechanisms with different polymeric materials and the degradation/dissolution rate of these composite membranes, versatile platforms can be developed, with optimum mechanical properties, drug/loading release kinetics and biological behavior. In a recent study, chitosan (CS) and polyethylene oxide (PEO) electrospun membranes loaded with MOX were investigated [99]. However, the authors reported the existence of MOX in the amorphous state in the membranes in contrast to the present study. In our study, the XRD patterns of the MOX-loaded neat PLGA membranes (30N2DL) indicated the existence of MOX in the semicrystalline phase. This is an indication that the drug was present in its crystalline state, perhaps due to aggregation issues and low dispersion of the drug in the polymeric solution. Further monitoring of dissolution conditions could yield optimized results [100,101]. This could also be related to the higher drug release rate of the MOX-loaded neat PLGA samples (30N2DL) compared to the composite samples (30A2DL) as the formation of drug crystals can affect drug distribution and release kinetics [101]. Hameed et al. [99] further evaluated the wound dressing, antibacterial activity, and healing efficacy, finding that MOX-loaded nanofibers presented greater stability, antibacterial properties, and wound healing efficiency compared to MOX-free membranes, confirming the effectiveness of MOX [99].

ISO10993-3 emphasizes the necessity of conducting hemocompatibility screening analysis for foreign materials when they come into contact with blood cells. Mass-dependent hemolytic-behavior of all the tested materials was observed indicating the sensitivity of erythrocytes when in contact with the PLGA-membranes. As previously reported [39,40,41,42,102], synthetic copolymers are hemocompatible under certain circumstances, and it is very important to consider all the parameters when designing implantable constructs. In the present study, MSN-loaded membranes presented slightly higher hemocompatibility compared with the neat, unloaded membranes; however, the differences were not statistically important, probably due to the small loading percentage of hemocompatible MSNs. Additionally, loading PLGA membranes with MOX presented reduced or no hemolytic activity, indicating the protective role of drugs/antibiotics when loaded into biomaterials, as previously reported for mesoporous nanocarriers loaded with artemisinin [103]. Further analysis in vivo could be considered to evaluate thrombosis and tissue interaction after a long time exposure to the materials. Further research is needed to evaluate the bioactivity and biocompatibiity of these scaffolds and their ability to induce differentiation of periodontal ligament cells towards osteogenic lineage, as well as their capability to regenerate periodontal tissues in vivo.

## 5. Conclusions

In this study, drug-loaded composite fibrous membranes (PLGA/MOX-MSNs) were produced by the electrospinning technique. Study of the polymer concentration and the synthesis conditions during the fabrication of the fibrous membranes revealed their effect on the size and morphology of the fibers. In particular, lower polymer concentration resulted in the formation of less uniform and indistinguishable fibers of smaller size. Study of the parameters during fiber formation showed that increase in the voltage led to increase in fiber diameter and the size of the pores. At the same time, increase in the collector speed fabricated membranes of slightly higher porosity. The use of MSNs as MOX nano-carriers in fibrous PLGA membranes seemed to improve the release of the drug by creating a more controlled and prolonged release profile and enhancing their hemolytic behavior, while the encapsulation of moxifloxacin presented a protective effect on the erythrocytes. In addition, it appears that increase in the polymer concentration yielded fibers with better dispersion of the MSNs, combining increased porosity and large fiber diameter. However, the addition of the MSNs limited the tensile and elongation strength. In addition, the rate of degradation and hydrophilicity increased with increasing MSNs amount. However, the encapsulation of the drug in membranes without the addition of MSNs was higher. Moreover, the addition of MSNs after encapsulation of the MOX did not change the morphology of the fibers, while the neat polymeric fibers, after the addition of the drug, led to a reduction in fiber cross-links. The proposed composite electrospun membranes could be an effective alternative strategy for the treatment of periodontal infections due to their controllable release of moxifloxacin ensuring antibacterial activity against an extensive variety of periodontal pathogens with a flexible degradation rate. Such barrier membranes could overcome the constraints related to hydrophobicity and restricted bioactivity and induce osteogenetic and angiogenic capacity.

## Figures and Tables

**Table 1:** Parameters of the electrospinning process for the synthesis of the PLGA-based electrospun membranes and calculated average fiber and pore diameters.

Sample	PLGA Concentration (% *w*/*v*)	MSNs Concentration (% *w*/*v*)	Voltage (kV)	Rotation Speed (rpm)	Average Fiber Diameter (nm)	Average Pore Diameter (μm)
20Ν1	20	-	22	1100	299	0.82
20Ν2	20	-	22	500	297	0.77
20Ν3	20	-	22	400	283	0.62
20Ν4	20	-	18	1100	278	0.62
20Ν5	20	-	18	500	239	0.54
20Ν6	20	-	18	400	239	0.54
30Ν1	30	-	22	1100	728	2.15
30Ν2	30	-	22	800	718	1.49
30Ν3	30	-	22	500	716	1.48
30Ν4	30	-	22	400	707	1.47
30Ν5	30	-	18	1100	767	1.55
30Ν6	30	-	18	500	759	1.50
30Ν7	30	-	18	400	759	1.47
20A1	20	10	22	1100	421	1.72
20A2	20	10	22	500	383	1.45
20A3	20	10	22	400	340	1.34
20A4	20	10	18	1100	335	1.31
20A5	20	10	18	500	315	1.14
20A6	20	10	18	400	312	1.11
30A1	30	10	22	1100	436	2.92
30A2	30	10	22	800	434	2.45
30A3	30	10	22	500	374	2.05
30A4	30	10	22	400	332	1.59
30A5	30	10	18	1100	274	1.77
30A6	30	10	18	500	214	1.28
30A7	30	10	18	400	211	1.20
30B	30	3	22	800	445	1.46

**Table 2:** Mechanical properties of the electrospun membranes (*n* = 10 samples per group).

	30N2	30A2	30B
UTS Stress (MPa)	3.8 ± 0.5	2.5 ± 0.2	1.2 ± 0.2
Tensile strain (%)	77.3 ± 12.7	18.8 ± 3.9	55.2 ± 13.7
Young’s modulus (MPa)	39.0 ± 0.2	33.9 ± 0.2	13.1 ± 0.1

**Table 3:** Drug content of the PLGA drug-loaded nanofibers and the PLGA nanofibers incorporating loaded MSNs.

Sample	Drug Loading (%)
30N2DL	37.2
30A2DL	27.5

## Data Availability

The data presented in this study are available on request from the corresponding author. The data are not publicly available due to privacy issues.

## References

[1] Martin-Cabezas R., Davideau J.-L., Tenenbaum H., Huck O. (2016). Clinical efficacy of probiotics as an adjunctive therapy to non-surgical periodontal treatment of chronic periodontitis: A systematic review and meta-analysis. J. Clin. Periodontol..

[2] Feres M., Figueiredo L.C., Soares G.M.S., Faveri M. (2015). Systemic antibiotics in the treatment of periodontitis. Periodontol. 2000.

[3] Karring T., Nyman S., Gottlow J.A.N., Laurell L. (1993). Development of the biological concept of guided tissue regeneration? Animal and human studies. Periodontology 2000.

[4] Bottino M.C., Thomas V., Schmidt G., Vohra Y.K., Chu T.-M.G., Kowolik M.J., Janowski G. (2012). Recent advances in the development of GTR/GBR membranes for periodontal regeneration—A materials perspective. Dent. Mater..

[5] Woo H.N., Cho Y.J., Tarafder S., Lee C.H. (2021). The recent advances in scaffolds for integrated periodontal regeneration. Bioact. Mater..

[6] Zhuang Y., Lin K., Yu H. (2019). Advance of Nano-Composite Electrospun Fibers in Periodontal Regeneration. Front Chem..

[7] Agrawal C.M., Ray R.B. (2001). Biodegradable polymeric scaffolds for musculoskeletal tissue engineering. J. Biomed. Mater. Res..

[8] Guo B., Ma P.X. (2014). Synthetic biodegradable functional polymers for tissue engineering: A brief review. Sci. China Chem..

[9] Balla E., Daniilidis V., Karlioti G., Kalamas T., Stefanidou M., Bikiaris N.D., Vlachopoulos A., Koumentakou I., Bikiaris D.N. (2021). Poly(lactic Acid): A Versatile Biobased Polymer for the Future with Multifunctional Properties—From Monomer Synthesis, Polymerization Techniques and Molecular Weight Increase to PLA Applications. Polymers.

[10] Nair L.S., Laurencin C.T. (2007). Biodegradable polymers as biomaterials. Prog. Polym. Sci..

[11] Turnbull G., Clarke J., Picard F., Riches P., Jia L., Han F., Li B., Shu W. (2018). 3D bioactive composite scaffolds for bone tissue engineering. Bioact. Mater..

[12] Vlachopoulos A., Karlioti G., Balla E., Daniilidis V., Kalamas T., Stefanidou M., Bikiaris N.D., Christodoulou E., Koumentakou I., Karavas E. (2022). Poly (Lactic Acid)-Based Microparticles for Drug Delivery Applications: An Overview of Recent Advances. Pharmaceutics.

[13] Gorth D., Webster T.J. (2011). Matrices for tissue engineering and regenerative medicine. Biomaterials for Artificial Organs.

[14] Sun X., Xu C., Wu G., Ye Q., Wang C. (2017). Poly(Lactic-co-Glycolic Acid): Applications and Future Prospects for Periodontal Tissue Regeneration. Polymers.

[15] Kyllönen L., D’Este M., Alini M., Eglin D. (2015). Local drug delivery for enhancing fracture healing in osteoporotic bone. Acta Biomater..

[16] Wei L., Ke J., Prasadam I., Miron R.J., Lin S., Xiao Y., Chang J., Wu C., Zhang Y. (2014). A comparative study of Sr-incorporated mesoporous bioactive glass scaffolds for regeneration of osteopenic bone defects. Osteoporos. Int..

[17] Sheikh I., Dahman Y. (2016). Applications of nanobiomaterials in hard tissue engineering. Nanobiomaterials in Hard Tissue Engineering.

[18] Nassar E.J., Neri C.R., Calefi P.S., Serra O.A. (1999). Functionalized silica synthesized by sol–gel process. J. Non-Cryst. Solids.

[19] Singh S., Chen H., Shahrokhi S., Wang L.P., Lin C.-H., Hu L., Guan X., Tricoli A., Xu Z.J., Wu T. (2020). Hybrid Organic–Inorganic Materials and Composites for Photoelectrochemical Water Splitting. ACS Energy Lett..

[20] Vrancken K., Possemiers K., Van Der Voort P., Vansant E. (1995). Surface modification of silica gels with aminoorganosilanes. Colloids Surfaces A Physicochem. Eng. Asp..

[21] Beari F., Brand M., Jenkner P., Lehnert R., Metternich H.J., Monkiewicz J., Siesler H.W. (2001). Organofunctional alkoxysilanes in dilute aqueous solution: New accounts on the dynamic structural mutability. J. Organomet. Chem..

[22] Nassar E.J., Ciuffi K.J., Ribeiro S.J.L., Messaddeq Y. (2003). Europium incorporated in silica matrix obtained by sol-gel: Luminescent materials. Mater. Res..

[23] Jackson C.L., Bauer B.J., Nakatani A.I., Barnes J.D. (1996). Synthesis of Hybrid Organic−Inorganic Materials from Interpenetrating Polymer Network Chemistry. Chem. Mater..

[24] Shea K.J., Loy D.A., Webster O. (1992). Arylsilsesquioxane gels and related materials. New hybrids of organic and inorganic networks. J. Am. Chem. Soc..

[25] Corriu R.J.P., Leclercq D. (1996). Recent Developments of Molecular Chemistry for Sol–Gel Processes. Angew. Chem. Int. Ed. Engl..

[26] Corriu R. (1998). A new trend in metal-alkoxide chemistry: The elaboration of monophasic organic-inorganic hybrid materials. Polyhedron.

[27] Cerveau G., Corriu R.J.P., Lepeytre C., Mutin P.H. (1998). Influence of the nature of the organic precursor on the textural and chemical properties of silsesquioxane materials. J. Mater. Chem..

[28] Pouroutzidou G.K., Liverani L., Theocharidou A., Tsamesidis I., Lazaridou M., Christodoulou E., Beketova A., Pappa C., Triantafyllidis K.S., Anastasiou A.D. (2021). Synthesis and Characterization of Mesoporous Mg- and Sr-Doped Nanoparticles for Moxifloxacin Drug Delivery in Promising Tissue Engineering Applications. Int. J. Mol. Sci..

[29] Landersdorfer C.B., Kinzig M., Hennig F.F., Bulitta J.B., Holzgrabe U., Drusano G.L., Sörgel F., Gusinde J. (2009). Penetration of Moxifloxacin into Bone Evaluated by Monte Carlo Simulation. Antimicrob. Agents. Chemother..

[30] Flemmig T.F., Petersilka G., Völp A., Gravemeier M., Zilly M., Mross D., Prior K., Yamamoto J., Beikler T. (2011). Efficacy and Safety of Adjunctive Local Moxifloxacin Delivery in the Treatment of Periodontitis. J. Periodontol..

[31] Milazzo I., Blandino G., Musumeci R., Nicoletti G., Lo Bue A., Speciale A. (2002). Antibacterial activity of moxifloxacin against periodontal anaerobic pathogens involved in systemic infections. Int. J. Antimicrob. Agents..

[32] Müller H.-P., Holderrieth S., Burkhardt U., Höffler U. (2002). In vitro antimicrobial susceptibility of oral strains of Actinobacillus actinomycetemcomitans to seven antibiotics. J Clin. Periodontol..

[33] Eick S., Seltmann T., Pfister W. (2004). Efficacy of antibiotics to strains of periodontopathogenic bacteria within a single species biofilm—An in vitro study. J. Clin. Periodontol..

[34] Guentsch A., Jentsch H., Pfister W., Hoffmann T., Eick S. (2008). Moxifloxacin as an Adjunctive Antibiotic in the Treatment of Severe Chronic Periodontitis. J. Periodontol..

[35] Eick S., Pfister W. (2004). Efficacy of Antibiotics Against Periodontopathogenic Bacteria Within Epithelial Cells: An In Vitro Study. J. Periodontol..

[36] Henkelman S., Rakhorst G., Blanton J., van Oeveren W. (2009). Standardization of incubation conditions for hemolysis testing of biomaterials. Mater. Sci. Eng. C.

[37] Albrektsson T., Johansson C. (2001). Osteoinduction, osteoconduction and osseointegration. Eur. Spine J..

[38] Cho Y.-D., Kim K.-H., Lee Y.-M., Ku Y., Seol Y.-J. (2021). Periodontal Wound Healing and Tissue Regeneration: A Narrative Review. Pharmaceuticals.

[39] Alehosseini M., Golafshan N., Kharaziha M., Fathi M., Edris H. (2018). Hemocompatible and Bioactive Heparin-Loaded PCL-α-TCP Fibrous Membranes for Bone Tissue Engineering. Macromol. Biosci..

[40] Cao C., Song Y., Yao Q., Yao Y., Wang T., Huang B., Gong P. (2015). Preparation and preliminary in vitro evaluation of a bFGF-releasing heparin-conjugated poly(ε-caprolactone) membrane for guided bone regeneration. J. Biomater. Sci. Polym. Ed..

[41] Shoba E., Lakra R., Kiran M.S., Korrapati P.S. (2020). 3D nano bilayered spatially and functionally graded scaffold impregnated bromelain conjugated magnesium doped hydroxyapatite nanoparticle for periodontal regeneration. J. Mech. Behav. Biomed. Mater..

[42] Padalhin A.R., Thuy Ba Linh N., Ki Min Y., Lee B.-T. (2014). Evaluation of the cytocompatibility hemocompatibility in vivo bone tissue regenerating capability of different PCL blends. J. Biomater. Sci. Polym. Ed..

[43] Maji K., Pramanik K. (2021). Electrospun scaffold for bone regeneration. Int. J. Polym. Mater. Polym. Biomater..

[44] Dzenis Y. (2004). Spinning Continuous Fibers for Nanotechnology. Science.

[45] Fong H., Chun I., Reneker D. (1999). Beaded nanofibers formed during electrospinning. Polymer.

[46] Venugopal J.R., Low S., Choon A.T., Kumar A.B., Ramakrishna S. (2008). Nanobioengineered Electrospun Composite Nanofibers and Osteoblasts for Bone Regeneration. Artif. Organs.

[47] Ding T., Li J., Zhang X., Du L., Li Y., Li D., Kong B., Ge S. (2020). Super-assembled core/shell fibrous frameworks with dual growth factors for in situ cementum–ligament–bone complex regeneration. Biomater. Sci..

[48] Cai X., Yang F., Yan X., Yang W., Yu N., Oortgiesen D.A.W., Wang Y., Jansen J.A., Walboomers X.F. (2015). Influence of bone marrow-derived mesenchymal stem cells pre-implantation differentiation approach on periodontal regeneration in vivo. J. Clin. Periodontol..

[49] Pham Q.P., Sharma U., Mikos A.G. (2006). Electrospinning of Polymeric Nanofibers for Tissue Engineering Applications: A Review. Tissue Eng..

[50] Teo W.E., Ramakrishna S. (2006). A review on electrospinning design and nanofibre assemblies. Nanotechnology.

[51] Unnithan A.R., Arathyram R.S., Kim C.S. (2015). Electrospinning of Polymers for Tissue Engineering. Nanotechnology Applications for Tissue Engineering.

[52] Barrientos I.J.H., Paladino E., Szabó P., Brozio S., Hall P.J., Oseghale C.I., Passarelli M.K., Moug S.J., Black R.A., Wilson C.G. (2017). Electrospun collagen-based nanofibres: A sustainable material for improved antibiotic utilisation in tissue engineering applications. Int. J. Pharm..

[53] Grafahrend D., Heffels K.-H., Beer M.V., Gasteier P., Möller M., Boehm G., Dalton P.D., Groll J. (2011). Degradable polyester scaffolds with controlled surface chemistry combining minimal protein adsorption with specific bioactivation. Nat. Mater..

[54] Li D., Xia Y. (2004). Electrospinning of Nanofibers: Reinventing the Wheel?. Adv. Mater..

[55] Yarin A.L., Koombhongse S., Reneker D.H. (2001). Taylor cone and jetting from liquid droplets in electrospinning of nanofibers. J. Appl. Phys..

[56] Reneker D.H., Chun I. (1996). Nanometre diameter fibres of polymer, produced by electrospinning. Nanotechnology.

[57] Doshi J., Reneker D.H. (1995). Electrospinning process and applications of electrospun fibers. J. Electrostat..

[58] Li H., Chang J. (2005). pH-compensation effect of bioactive inorganic fillers on the degradation of PLGA. Compos. Sci. Technol..

[59] Hu C., Liu S., Zhang Y., Li B., Yang H., Fan C., Cui W. (2013). Long-term drug release from electrospun fibers for in vivo inflammation prevention in the prevention of peritendinous adhesions. Acta Biomater..

[60] Song B., Wu C., Chang J. (2012). Dual drug release from electrospun poly(lactic-co-glycolic acid)/mesoporous silica nanoparticles composite mats with distinct release profiles. Acta Biomater..

[61] Shin H.J., Lee C.H., Cho I.H., Kim Y.-J., Lee Y.-J., Kim I.A., Park K.-D., Yui N., Shin J.-W. (2006). Electrospun PLGA nanofiber scaffolds for articular cartilage reconstruction: Mechanical stability, degradation and cellular responses under mechanical stimulation in vitro. J. Biomater. Sci. Polym. Ed..

[62] Lu L., Peter S.J., Lyman M.D., Lai H.-L., Leite S.M., Tamada J.A., Uyama S., Vacanti J.P., Langer R., Mikos A.G. (2000). In vitro and in vivo degradation of porous poly(dl-lactic-co-glycolic acid) foams. Biomaterials.

[63] Lu L., Garcia C.A., Mikos A.G. (1999). In vitro degradation of thin poly(DL-lactic-co-glycolic acid) films. J. Biomed. Mater. Res..

[64] Paragkumar N.T., Edith D., Six J.-L. (2006). Surface characteristics of PLA and PLGA films. Appl. Surf. Sci..

[65] Akl M.A., Kartal-Hodzic A., Oksanen T., Ismael H.R., Afouna M.M., Yliperttula M., Samy A.M., Viitala T. (2016). Factorial design formulation optimization and in vitro characterization of curcumin-loaded PLGA nanoparticles for colon delivery. J. Drug Deliv. Sci. Technol..

[66] Jia Y., Zhang H., Yang S., Xi Z., Tang T., Yin R., Zhang W. (2018). Electrospun PLGA membrane incorporated with andrographolide-loaded mesoporous silica nanoparticles for sustained antibacterial wound dressing. Nanomedicine.

[67] Al Omari M.M.H., Jaafari D.S., Al-Sou’od K.A., Badwan A.A. (2014). Moxifloxacin Hydrochloride. Profiles of Drug Substances, Excipients and Related Methodology.

[68] Nagarjuna Reddy Y., Deepika P., Venkatesh M., Rajeshwari K. (2016). Evaluation of moxifloxacin-hydroxyapatite composite graft in the regeneration of intrabony defects: A clinical, radiographic, and microbiological study. Contemp. Clin. Dent..

[69] Li Z., Clemens D.L., Lee B.-Y., Dillon B.J., Horwitz M.A., Zink J.I. (2015). Mesoporous Silica Nanoparticles with pH-Sensitive Nanovalves for Delivery of Moxifloxacin Provide Improved Treatment of Lethal Pneumonic Tularemia. ACS Nano.

[70] Lee B., Li Z., Clemens D.L., Dillon B.J., Hwang A.A., Zink J.I., Horwitz M.A. (2016). Redox-Triggered Release of Moxifloxacin from Mesoporous Silica Nanoparticles Functionalized with Disulfide Snap-Tops Enhances Efficacy Against Pneumonic Tularemia in Mice. Small.

[71] Chen Y., Zhou S., Li Q. (2011). Mathematical modeling of degradation for bulk-erosive polymers: Applications in tissue engineering scaffolds and drug delivery systems. Acta Biomater..

[72] Júlio T.A., Garcia J.S., Bonfilio R., Araújo M.B., Trevisan M.G. (2015). Solid-state stability and solubility determination of crystalline forms of moxifloxacin hydrochloride. Int. J. Pharm. Pharm. Sci..

[73] Filippousi M., Siafaka P.I., Amanatiadou E.P., Nanaki S.G., Nerantzaki M., Bikiaris D.N., Vizirianakis I.S., Van Tendeloo G. (2015). Modified chitosan coated mesoporous strontium hydroxyapatite nanorods as drug carriers. J. Mater. Chem. B.

[74] Pham Q.P., Sharma U., Mikos A.G. (2006). Electrospun Poly(ε-caprolactone) Microfiber and Multilayer Nanofiber/Microfiber Scaffolds: Characterization of Scaffolds and Measurement of Cellular Infiltration. Biomacromolecules.

[75] Mousavi S.-M., Nejad Z.M., Hashemi S.A., Salari M., Gholami A., Ramakrishna S., Chiang W.-H., Lai C.W. (2021). Bioactive Agent-Loaded Electrospun Nanofiber Membranes for Accelerating Healing Process: A Review. Membranes.

[76] Rnjak-Kovacina J., Weiss A.S. (2011). Increasing the Pore Size of Electrospun Scaffolds. Tissue Eng. Part B Rev..

[77] Lowery J.L., Datta N., Rutledge G.C. (2010). Effect of fiber diameter, pore size and seeding method on growth of human dermal fibroblasts in electrospun poly(ɛ-caprolactone) fibrous mats. Biomaterials.

[78] Nam J., Huang Y., Agarwal S., Lannutti J. (2007). Improved Cellular Infiltration in Electrospun Fiber via Engineered Porosity. Tissue Eng..

[79] Soliman S., Pagliari S., Rinaldi A., Forte G., Fiaccavento R., Pagliari F., Franzese O., Minieri M., Di Nardo P., Licoccia S. (2010). Multiscale three-dimensional scaffolds for soft tissue engineering via multimodal electrospinning. Acta Biomater..

[80] Khil M.-S., Cha D.-I., Kim H.-Y., Kim I.-S., Bhattarai N. (2003). Electrospun nanofibrous polyurethane membrane as wound dressing. J. Biomed. Mater. Res..

[81] Van Tienen T.G., Heijkants R.G.J., Buma P., de Groot J.H., Pennings A.J., Veth R.P. (2002). Tissue ingrowth and degradation of two biodegradable porous polymers with different porosities and pore sizes. Biomaterials.

[82] Sisson K., Zhang C., Farach-Carson M.C., Chase D.B., Rabolt J.F. (2010). Fiber diameters control osteoblastic cell migration and differentiation in electrospun gelatin. J. Biomed. Mater. Res. Part A.

[83] Rnjak J., Li Z., Maitz P.K.M., Wise S.G., Weiss A.S. (2009). Primary human dermal fibroblast interactions with open weave three-dimensional scaffolds prepared from synthetic human elastin. Biomaterials.

[84] Rnjak-Kovacina J., Wise S.G., Li Z., Maitz P.K.M., Young C.J., Wang Y., Weiss A.S. (2011). Tailoring the porosity and pore size of electrospun synthetic human elastin scaffolds for dermal tissue engineering. Biomaterials.

[85] Balguid A., Mol A., van Marion M.H., Bank R.A., Bouten C.V.C., Baaijens F.P.T. (2009). Tailoring Fiber Diameter in Electrospun Poly(ɛ-Caprolactone) Scaffolds for Optimal Cellular Infiltration in Cardiovascular Tissue Engineering. Tissue Eng. Part A.

[86] Powell D.W., Mifflin R.C., Valentich J.D., Crowe S.E., Saada J.I., West A.B. (1999). Myofibroblasts. I. Paracrine cells important in health and disease. Am. J. Physiol. Physiol..

[87] Eichhorn S.J., Sampson W.W. (2005). Statistical geometry of pores and statistics of porous nanofibrous assemblies. J. R. Soc. Interface.

[88] Motamedi A.S., Mirzadeh H., Hajiesmaeilbaigi F., Bagheri-Khoulenjani S., Shokrgozar M. (2017). Effect of electrospinning parameters on morphological properties of PVDF nanofibrous scaffolds. Prog. Biomater..

[89] Alhamdani G.M., Al-Turaihi B.A., Al-Masoody A.H. (2019). Electrospinning approaches for periodontal regeneration: A review. Drug Invent. Today..

[90] Zargham S., Bazgir S., Tavakoli A., Rashidi A.S., Damerchely R. (2012). The Effect of Flow Rate on Morphology and Deposition Area of Electrospun Nylon 6 Nanofiber. J. Eng. Fiber Fabr..

[91] Yuan X., Zhang Y., Dong C., Sheng J. (2004). Morphology of ultrafine polysulfone fibers prepared by electrospinning. Polym. Int..

[92] Liu Y., Dong L., Fan J., Wang R., Yu J.-Y. (2011). Effect of applied voltage on diameter and morphology of ultrafine fibers in bubble electrospinning. J. Appl. Polym. Sci..

[93] Demir M., Yilgor I., Yilgor E., Erman B. (2002). Electrospinning of polyurethane fibers. Polymer.

[94] Zare Y. (2016). Study of nanoparticles aggregation/agglomeration in polymer particulate nanocomposites by mechanical properties. Compos. Part A Appl. Sci. Manuf..

[95] Heggannavar G.B., Vijeth S., Kariduraganavar M.Y. (2019). Development of dual drug loaded PLGA based mesoporous silica nanoparticles and their conjugation with Angiopep-2 to treat glioma. J. Drug Deliv. Sci. Technol..

[96] Kikuchi M., Koyama Y., Yamada T., Imamura Y., Okada T., Shirahama N., Akita K., Takakuda K., Tanaka J. (2004). Development of guided bone regeneration membrane composed of β-tricalcium phosphate and poly (l-lactide-co-glycolide-co-ε-caprolactone) composites. Biomaterials.

[97] Wang J., Wang L., Zhou Z., Lai H., Xu P., Liao L., Wei J. (2016). Biodegradable Polymer Membranes Applied in Guided Bone/Tissue Regeneration: A Review. Polymers.

[98] Xue J., He M., Liang Y., Crawford A., Coates P., Chen D., Shi R., Zhang L. (2014). Fabrication and evaluation of electrospun PCL–gelatin micro-/nanofiber membranes for anti-infective GTR implants. J. Mater. Chem. B.

[99] Hameed M., Rasul A., Nazir A., Yousaf A.M., Hussain T., Khan I.U., Abbas G., Abid S., Yousafi Q.U.A., Ghori M.U. (2021). Moxifloxacin-loaded electrospun polymeric composite nanofibers-based wound dressing for enhanced antibacterial activity and healing efficacy. Int. J. Polym. Mater. Polym. Biomater..

[100] Luraghi A., Peri F., Moroni L. (2021). Electrospinning for drug delivery applications: A review. J. Control. Release.

[101] Seif S., Franzen L., Windbergs M. (2015). Overcoming drug crystallization in electrospun fibers—Elucidating key parameters and developing strategies for drug delivery. Int. J. Pharm..

[102] Ammann K.R., Hossainy S.F.A., Hossainy S., Slepian M.J. (2021). Hemocompatibility of polymers for use in vascular endoluminal implants. J. Appl. Polym. Sci..

[103] Tsamesidis I., Gkiliopoulos D., Pouroutzidou G.K., Lymperaki E., Papoulia C., Reybier K., Perio P., Paraskevopoulos K.M., Kontonasaki E., Theocharidou A. (2021). Effect of Artemisinin-Loaded Mesoporous Cerium-Doped Calcium Silicate Nanopowder on Cell Proliferation of Human Periodontal Ligament Fibroblasts. Nanomaterials.

